# Gingipain-Mediated Matrix Metalloproteinase-8 and -9 Activation in Oral Rinse Samples from Patients with Cancer: A Cross-Sectional Study

**DOI:** 10.3390/dj14050272

**Published:** 2026-05-06

**Authors:** Nur Rahman Ahmad Seno Aji, Sebastian K. Ahlstrand, Vaibhav Sahni, Kartika W. Taroeno-Hariadi, Dyah Listyarifah, Rezmelia Sari, Iffah Mardhiyah, Ahmad Syaify, Asikin Nur, Aditya Lia Ramadona, Julie Toby Thomas, Tommi Pätilä, Antti A. Mäkitie, Ismo T. Räisänen, Pirjo Pärnänen, Pietro Leone, Sukumaran Anil, Timo Sorsa

**Affiliations:** 1 Department of Oral and Maxillofacial Diseases, Faculty of Medicine, University of Helsinki, Haartmaninkatu 8, 00014 Helsinki, Finland; julie.tobythomas@helsinki.fi (J.T.T.); ismo.raisanen@helsinki.fi (I.T.R.); pirjo.parnanen@helsinki.fi (P.P.); timo.sorsa@helsinki.fi (T.S.); 2 Department of Periodontics, Faculty of Dentistry, Universitas Gadjah Mada, Yogyakarta 55281, Indonesia; rezmelia.sari@mail.ugm.ac.id (R.S.); ahmad.syaify@ugm.ac.id (A.S.); 3 Department of Otorhinolaryngology, Head and Neck Surgery, University of Helsinki and Helsinki University Hospital, Kasarmikatu 11-23, 00029 Helsinki, Finland; sebastian.ahlstrand@helsinki.fi (S.K.A.); antti.makitie@hus.fi (A.A.M.); 4 All India Institute of Medical Sciences, New Delhi 110029, India; vaibhav.sahni@rcsed.net; 5 Divison of Hematology-Medical Oncology, Department of Internal Medicine, Faculty of Medicine, Public Health and Nursing, Dr Sardjito Hospital, Universitas Gadjah Mada, Yogyakarta 55281, Indonesia; kartika.widayati@ugm.ac.id; 6 Department of Dental Biomedical Sciences, Faculty of Dentistry, Universitas Gadjah Mada, Yogyakarta 55281, Indonesia; dlistyarifah@ugm.ac.id (D.L.); asikin@mail.ugm.ac.id (A.N.); 7 Department of Conservative Dentistry, Faculty of Dentistry, Universitas Gadjah Mada, Yogyakarta 55281, Indonesia; iffahmardhiyah@ugm.ac.id; 8 Dental Hygiene Program, Faculty of Dentistry, Universitas Gadjah Mada, Yogyakarta 55281, Indonesia; 9 Department of Health Behaviour, Environment, and Social Medicine, Faculty of Medicine, Public Health and Nursing, Universitas Gadjah Mada, Yogyakarta 55281, Indonesia; alramadona@ugm.ac.id; 10 Department of Periodontics, Saveetha Dental College and Hospitals, Saveetha Institute of Medical and Technical Sciences, Chennai 600077, India; 11 Department of Congenital Heart Surgery and Organ Transplantation, New Children’s Hospital, University of Helsinki, 00290 Helsinki, Finland; tommi.patila@hus.fi; 12 Research Program in Systems Oncology, Faculty of Medicine, University of Helsinki, Haartmaninkatu 8, 00014 Helsinki, Finland; 13 Division of Ear, Nose and Throat Diseases, Department of Clinical Sciences, Intervention and Technology, Karolinska Institute and Karolinska University Hospital, 171 76 Stockholm, Sweden; 14 SmileLongevityCare360 Centre, 80122 Naples, Italy; pietro_leone@hotmail.it; 15 Faculty of Dentistry, Chulalongkorn University, Pathumwan, Bangkok 10330, Thailand; drsanil@gmail.com; 16 Global Research Cell, Dr. D. Y. Patil Dental College and Hospital, Dr. D. Y. Patil Vidyapeeth, Pune 411018, India; 17 Division of Oral Diseases, Department of Dental Medicine, Karolinska Institutet, 171 77 Stockholm, Sweden

**Keywords:** cancer screening, aMMP-8, MMP-9, gingipain, oral rinse biomarker

## Abstract

**Objectives**: To investigate whether *Porphyromonas gingivalis* gingipains contribute to the activation of MMP-8 and MMP-9 in patients with cancer and to evaluate the potential of aMMP-8 chair-side test for early detection of active periodontal inflammation in cancer care. **Methods**: This cross-sectional study enrolled 89 of 119 initially assessed patients with cancer at Dr. Sardjito Hospital, Yogyakarta, Indonesia (2023–2024). Active MMP-8 was quantified using a chair-side test on oral rinse samples. Total MMP-8 was measured by ELISA, MMP-9 activation by gelatin zymography, and gingipain presence by Western immunoblotting. Associations with periodontal parameters (PPD, GI, OHI-S, and VPI) were analyzed using linear regression. **Results**: aMMP-8 chair-side test demonstrated 91% positive results in 89 patients with cancer. Western blotting revealed the co-localization of gingipains with fragmented MMP-8. aMMP-8 levels were significantly associated with MMP-9 activation (*p* = 0.003, R^2^ = 0.097), suggesting proteolytic cascade activation. Oral hygiene indices showed stronger associations (OHI-S: *p* = 0.003, R^2^ = 0.100; VPI: *p* = 0.014, R^2^ = 0.067) than deeper periodontal parameters (PPD: *p* = 0.094; GI: *p* = 0.358), suggesting early-stage inflammation detection. **Conclusions**: Gingipain presence may be associated with the activation of MMP-8 and MMP-9 in patients with cancer, which links bacterial virulence to amplified tissue destruction. aMMP-8 chair-side test enables rapid identification of active inflammation, facilitating timely periodontal intervention during cancer care.

## 1. Introduction

GLOBOCAN 2022 data suggest a cancer incidence of over 19 million, with 5-year prevalence at about 53.5 million cases and approximately 9.7 million mortalities attributable to cancer [1]. Periodontitis disproportionately affects patients with cancer, with prevalence reaching 69.5% in colorectal cancer [2] and 68% in nasopharyngeal cancer requiring tooth extraction [3]. This elevated burden stems from both cancer-related immunosuppression and treatment-induced oral complications. While periodontal therapy improves clinical parameters in healthy populations [4], patients with cancer exhibit diminished treatment responses, as reflected by persistent residual periodontal pockets and limited reduction in CRP levels following scaling and root planing [5,6].

The molecular mechanisms driving accelerated periodontal destruction in patients with cancer remain poorly understood. Matrix metalloproteinase-8 (MMP-8), the primary collagenase in periodontal tissues, exists in latent and activated forms. Only activated MMP-8 (aMMP-8) reflects ongoing tissue breakdown, suggesting its potential as a valuable biomarker compared to conventional assessments that merely document past damage [7]. A chair-side test for aMMP-8 enables rapid, non-invasive detection of active inflammation through lateral-flow immunoassay technology, offering significant advantages over traditional periodontal probing and radiography [8,9].

A critical knowledge gap exists regarding the interplay between bacterial proteases and host MMPs in patients with cancer. *Porphyromonas gingivalis* gingipains, cysteine proteases that activate latent MMPs and degrade protease inhibitors, may orchestrate a proteolytic cascade amplifying tissue destruction [10]. While individual MMP activation by gingipains has been demonstrated in vitro, clinical evidence of this cascade in patients with cancer is lacking [11].

We hypothesized that gingipains drive concurrent activation of MMP-8 and MMP-9 in patients with cancer, creating a proteolytic cascade that accelerates periodontal destruction. Our primary objective was to demonstrate associations between aMMP-8 chair-side test results and MMP-9 activation in oral rinse samples. Secondary objectives included correlating aMMP-8 with clinical parameters (PPD, GI, OHI-S, and VPI) and providing molecular evidence for gingipain involvement through Western immunoblotting and zymography.

## 2. Materials and Methods

### 2.1. Study Design and Participants

**Figure 1:**
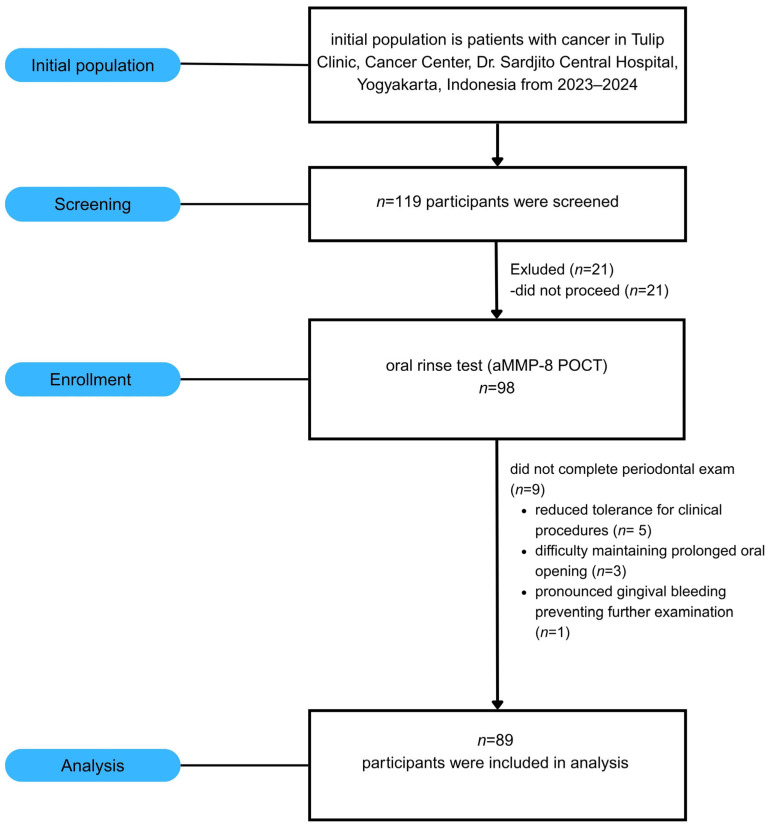
STROBE flowchart of participant selection and enrollment. Of 119 patients with cancer screened at the Tulip Cancer Clinic (2023–2024), 89 met the inclusion criteria and were enrolled for analysis.

This cross-sectional study (October 2023–March 2024) was conducted at the Tulip Cancer Clinic, Dr. Sardjito Hospital, Yogyakarta, Indonesia (Ethics: KE/FK/0071/EC/2023) in accordance with the Declaration of Helsinki. A sample size calculation indicated that 85 participants were needed to detect moderate correlations (r = 0.30) with 80% power at α = 0.05. Inclusion criteria comprised adults over 20 years of age with a confirmed cancer diagnosis who provided written informed consent. Patients were excluded if they reported a history of antibiotic therapy for the past six months, an active infectious disease, and were unable to undergo oral examination. Of the 119 patients initially assessed, 89 met these criteria and were enrolled in this study (Figure 1).

### 2.2. aMMP-8 Chair-Side Test

Participants were instructed to abstain from toothbrushing and eating for at least 1 h prior to sampling. Each participant first performed a 30 s rinse with tap water to remove oral debris, and this sample was discarded. After a 60 s interval, a second 30 s rinse was performed using 5 mL of *aqua purificata* (sterile distilled water) provided in the aMMP-8 chair-side test kit. This second rinse was collected for immediate determination of aMMP-8 levels using a lateral-flow immunoassay (PerioSafe^®^ Pro DRS, Dentognostics GmbH, Jena, Germany) and quantified with a portable photometric reader (ORALyzer^®^, Dentognostics GmbH, Jena, Germany) with a cutoff of 20 ng/mL. For this study, “positivity or positive result” was defined as any aMMP-8 level above the 20 ng/mL cutoff, corresponding to a positive reading (appearance of two lines) on the kit’s visual scale, including low [+], medium [++], and strong [+++] categories. The appearance of only one line was interpreted as a negative result. The sensitivity and specificity of the assay range from 76 to 90%. The remaining samples were stored at −70 °C for further analyses.

### 2.3. Periodontal Clinical Examination

Periodontal health status was comprehensively assessed according to the 2017 AAP consensus criteria, with periodontitis defined as bleeding on probing (BOP) at ≥10% of sites and probing pocket depth (PPD) > 3 mm. Clinical indices evaluated included the Gingival Index [12], Visible Plaque Index (VPI) [13], and Oral Hygiene Index-Simplified [14]. A single trained periodontist (R.S.) performed all examinations to ensure consistency and reliability. PPD was measured using a UNC15 periodontal probe (Osung, Houston, TX, USA) at six sites per tooth across all sextants. Additionally, the number of missing and mobile teeth was recorded.

### 2.4. Total MMP-8 ELISA

Total MMP-8 concentrations in oral rinse samples were quantified using a commercial ELISA kit (R&D Systems, Minneapolis, MN, USA) with a detection limit of 0.004–0.058 ng/mL, as validated by Umeizudike et al. [15]. The ELISA kit uses a polyclonal antibody approach to detect human total MMP-8, including latent (proMMP-8), active, and Tissue Inhibitor of Matrix metalloproteinases (TIMP)-complexed forms, without distinguishing among them. Samples were diluted at 1:10 and processed according to the manufacturer’s protocol: 2 h sample incubation, washing (4×), 2 h conjugate incubation, washing (4×), 30 min substrate incubation in darkness, and stop solution addition. Absorbance was measured at 450 nm using a Victor X plate reader (PerkinElmer, New York City, NY, USA). Total MMP-8 concentrations were calculated from standard curves and expressed as ng/mL.

### 2.5. MMP-9 Activation Zymography

Gelatin zymography was used to assess MMP-9 activation, following established protocols [16,17]. Oral rinse samples mixed with loading buffer were resolved on gelatin-copolymerized SDS-PAGE gels under controlled conditions (maintained at 4 °C, protected from light). After electrophoresis, gels were washed to remove SDS, incubated overnight at 37 °C in activity buffer, and then stained with Coomassie Blue. Proteolytic bands were imaged using a GS-700 densitometer (Bio-Rad, Hercules, CA, USA) and analyzed with Quantity One software version 4.6.8. MMP-9 activation was calculated as the ratio of active (82 kDa) to total (82 kDa + 92 kDa) band intensities.

### 2.6. Western Immunoblotting

A qualitative/semi-quantitative Western immunoblot was performed to detect the expression of MMP-8 and *P. gingivalis* gingipain. Samples were denatured (100 °C, 5 min), separated by SDS-PAGE, and transferred to nitrocellulose membranes. After overnight blocking at 4 °C (Intercept™ Buffer, LI-COR Biosciences, Lincoln, NE, USA), membranes were probed with rabbit polyclonal antibodies: anti-MMP-8 (A38881, antibodies.com) for MMP-8 detection and anti-gingipain (orb243611, Biorbyt) for *P. gingivalis* gingipains. Detection employed IR Dye-conjugated secondary antibodies (680LT and 800CW goat anti-rabbit, LI-COR) with imaging on the Odyssey Infrared System. Band intensities were analyzed using Image Studio v4.0 [18,19].

### 2.7. Statistical Analysis

Statistical analyses were performed in R (version 4.2.2) using the rstatix package (version 0.7.1). Linear regression examined associations between quantified aMMP-8 (ng/mL) and the percentage of MMP-9 activation, as well as between aMMP-8 and clinical periodontal parameters (PPD, GI, OHI-S, and VPI). The Mann–Whitney U-test was used to compare MMP-9 activation between patients with cancer and controls. Descriptive statistics (mean ± SD, frequencies, and percentages) were used to summarize demographic, clinical, and biomarker data. Statistical significance was set at *p* < 0.05. Data visualization utilized BioRender.

## 3. Results

### 3.1. Study Population Characteristics

Of 119 patients with cancer assessed (2023–2024), 89 met the inclusion criteria and were enrolled for analysis. The cohort was predominantly female (74.2%) with a mean age of 54.8 years (range 22–80). Cancer distribution comprised: breast (40.5%), nasopharyngeal (38.2%), colorectal (12.4%), lymphoma (4.5%), cervical (2.3%), lung (1.1%), and renal cancer (1.1%). Stratification by anatomical location revealed 38.2% head and neck cancers versus 61.8% non-head and neck malignancies (Table 1).

Treatment status at enrollment varied: chemotherapy (44.9%), combined chemoradiotherapy (20.2%), pre-treatment (16.9%), hormone therapy (8.9%), maintenance (8.9%), radiotherapy alone (3.4%), and immunotherapy (1.1%). Smoking prevalence was 6.7%. Oral hygiene practices in the cancer cohort varied, with 87.6% of participants reporting brushing twice daily, 12.4% brushing once daily, and none reporting no toothbrushing (Table 1).

### 3.2. Clinical Periodontal Parameters

Periodontal assessment revealed moderate to severe disease burden across the cohort. Mean probing pocket depth was 4.01 ± 1.32 mm, exceeding the periodontitis threshold. Oral hygiene indices indicated substantial plaque accumulation: OHI-S, 3.08 ± 0.90, and VPI, 2.53 ± 0.59. Gingival inflammation was present but relatively mild (GI 0.64 ± 0.46) compared to the plaque burden (Table 2).

### 3.3. aMMP-8 Chair-Side Test’s Performance and Validation

**Figure 2:**
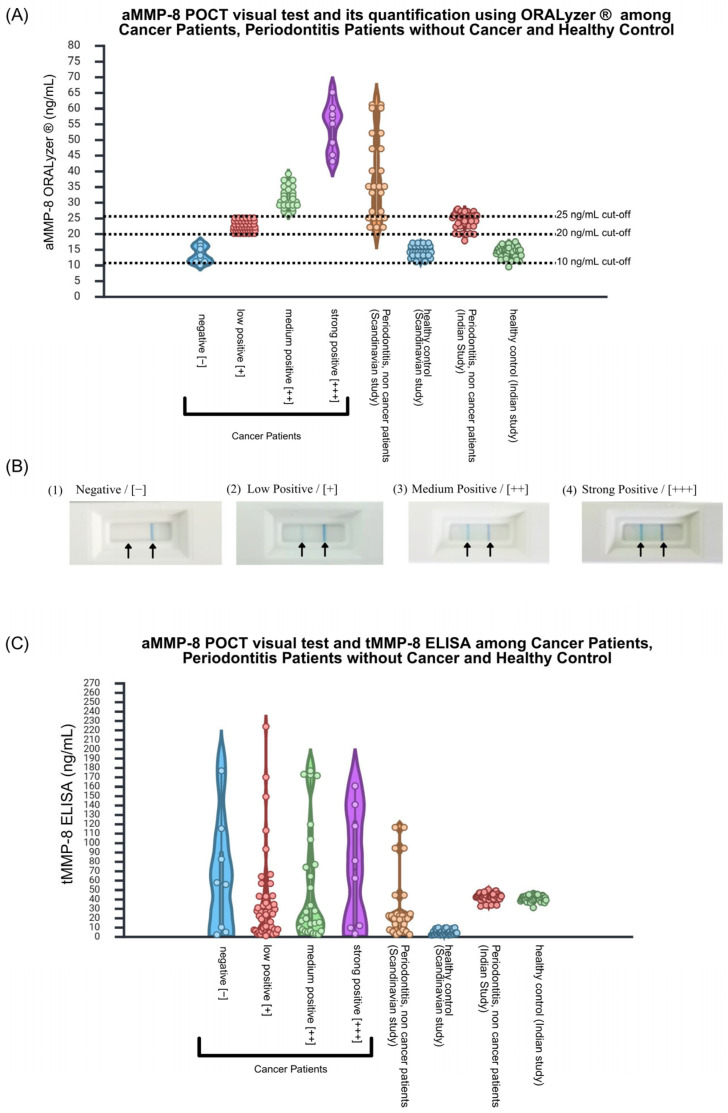
aMMP-8 chair-side test validation in patients with cancer. (**A**) Violin plot showing quantitative aMMP-8 concentrations (ng/mL) stratified by visual categories, demonstrating stepwise increases from negative to strong positive. Reference data from Scandinavian and Asian cohorts were included for comparison. (**B**) Representative chair-side test visual results: (**1**) negative, (**2**) low positive, (**3**) medium positive, and (**4**) strong positive. (**C**) Comparison of total versus active MMP-8 in patients with cancer. Violin plot showing total MMP-8 concentrations (ELISA, ng/mL) stratified by the aMMP-8 chair-side test’s visual categories. Note the lack of correlation between total MMP-8 levels and visual positivity categories, with comparable concentrations across the negative, low-positive, medium-positive, and strong-positive groups. Reference data from Scandinavian and Asian cohorts were included for comparison.

Visual aMMP-8 chair-side test demonstrated 91% positivity among patients with cancer, with the following distribution of different graded positivity according to Aji et al. [20]: negative 9.0%, low positive [+] 51.7%, medium positive [++] 30.3%, and strong positive [++] 9.0%. Quantitative validation using a photometric reader with software aLF Reader version 1.1.1 15478 (ORALyzer^®^) revealed a clear dose–response relationship across visual categories. Mean aMMP-8 concentrations increased stepwise: negative (≤20 ng/mL), low positive (20–30 ng/mL), medium positive (30–50 ng/mL), and strong positive (50–60 ng/mL), with an overall mean of 27.04 ± 10.55 ng/mL. This stepwise increase confirmed the reliability of visual interpretation (Figure 2A,B). Comparison with reference cohorts revealed that the aMMP-8 levels of patients with cancer exceeded those of systemically healthy controls but remained below the levels reported in severe periodontitis in Scandinavian [21] and Asian populations [22], suggesting an intermediate inflammatory status.

### 3.4. Total MMP-8 Lacks Diagnostic Discrimination

Total MMP-8 measured by ELISA averaged 46.38 ± 54.14 ng/mL across all patients. As presented in Figure 2C, the total MMP-8 assay does discriminate between visual chair-side test categories as active MMP-8 quantification by ORALyzer^®^, confirming that the two assays measure biologically distinct entities. While active MMP-8 showed a clear dose–response pattern, total MMP-8 concentrations showed no correlation with visual chair-side test results’ categories. Patients testing negative exhibited comparable total MMP-8 levels to those classified as low, medium, or strong positive, suggesting that only the activated form provides diagnostic value. This finding aligns with reference cohorts from Scandinavian and Indian populations [21,22], where total MMP-8 similarly failed to discriminate disease severity.

### 3.5. MMP-9 Activation and Proteolytic Activation Cascade Evidence

**Figure 3:**
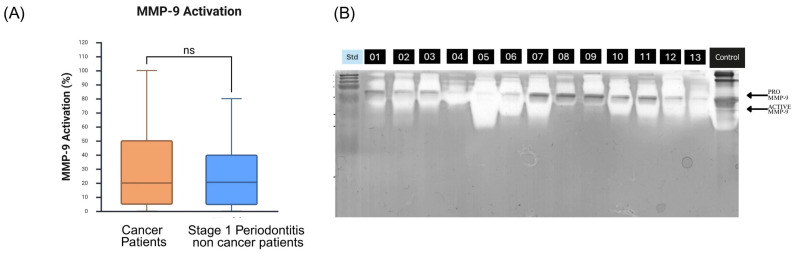
MMP-9 activation in patients with cancer demonstrates a proteolytic cascade. (**A**) Comparison of MMP-9 activation percentage between patients with cancer (n = 89) and healthy controls, showing elevated activation in the cancer cohort (29.15 ± 28.42% vs. 18.3 ± 15.2%, *p* = 0.688), ns= non significant. (**B**) Representative gelatin zymogram showing MMP-9 activation patterns. Lanes 1–13: oral rinse samples from patients with cancer displaying 92 kDa (latent MMP-9) and 82 kDa (active MMP-9) bands. Lane 14: positive control from a patient with periodontitis.

Gelatin zymography revealed a mean MMP-9 activation of 29.15 ± 28.42%, calculated as the ratio of active (82 kDa) to total (82 kDa + 92 kDa) band densities [16,17]. While patients with cancer showed higher MMP-9 activation compared to healthy controls from the external reference cohort (29.15% vs. 18.3%) [15], this difference was not statistically significant (Mann–Whitney U-test, *p* = 0.688) (Figure 3).

### 3.6. aMMP-8 Correlation with MMP-9 and Periodontal Clinical Parameter

**Figure 4:**
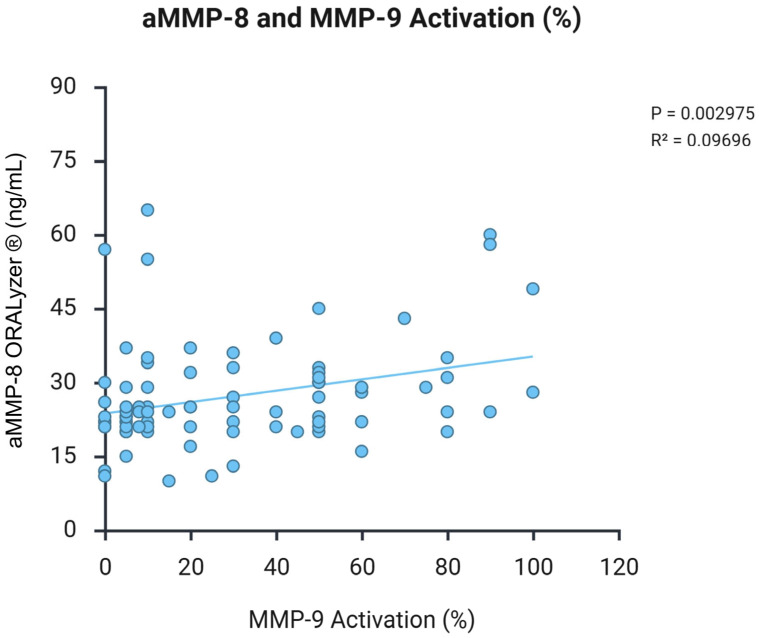
Correlation between aMMP-8 and MMP-9 activation, demonstrating a proteolytic cascade.

Scatter plots in Figure 4 show a significant positive association between aMMP-8 concentrations (ng/mL) and MMP-9 activation percentage in patients with cancer (n = 89). Linear regression analysis revealed *p* = 0.003 and R^2^ = 0.097, supporting coordinated proteolytic activity. Each point represents an individual patient sample.

**Figure 5:**
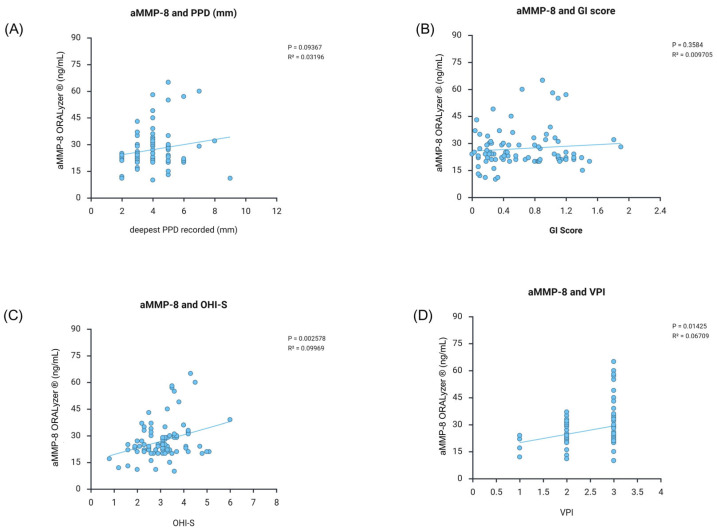
aMMP-8 is related to clinical periodontal parameters. (**A**). aMMP-8 shows a tendency to be associated with PPD, though not significantly. (**B**). aMMP-8 shows a tendency to be associated with GI, though not significantly. (**C**). aMMP-8 was significantly associated with OHI-S. (**D**) aMMP-8 was significantly associated with VPI.

aMMP-8 levels showed differential correlations with periodontal indices. Significant associations were observed with oral hygiene indices: OHI-S (*p* = 0.003, R^2^ = 0.100) and VPI (*p* = 0.014, R^2^ = 0.067). In contrast, deeper periodontal parameters showed positive trends, although they did not reach statistical significance: PPD (*p* = 0.094, R^2^ = 0.032) and GI (*p* = 0.358, R^2^ = 0.010). This pattern, stronger correlation with oral hygiene indices versus deeper tissue parameters, suggests that aMMP-8 primarily reflects early inflammatory responses to bacterial biofilm rather than established tissue destruction (Figure 5).

### 3.7. Molecular Evidence for Gingipain-Mediated Cascade

Qualitative western immunoblot analysis provided molecular evidence for the proteolytic cascade. Anti-MMP-8 antibody detected primary bands at approximately 70–80 kDa (likely representing MMP-8 complexes or glycosylated neutrophil MMP-8), with progressive fragmentation correlating with the chair-side test’s visual intensity. Negative samples showed intact MMP-8, while strong positive [+++] samples exhibited extensive degradation products (20–50 kDa bands), indicating step-wise proteolytic activation.

Anti-gingipain antibody against *P. gingivalis* detected bacterial protease in all positive samples. Signal intensity directly paralleled both visual positivity and MMP-8 fragmentation severity, with the strongest signals in [+++] samples.

**Figure 6:**
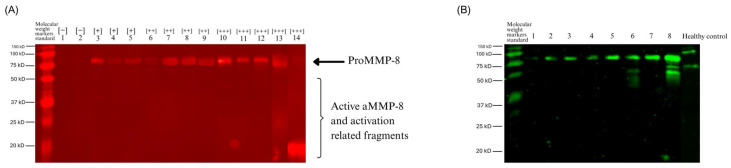
Western immunoblot analysis of the gingipain-mediated MMP-8 fragmentation cascade. (**A**) MMP-8 immunoblot shows fragmentation patterns corresponding to aMMP-8 chair-side test categories (−, +, ++, +++). Primary bands at 70–80 kDa likely represent glycosylated or modified pro-MMP-8, while active MMP-8 appears at 50–54 kDa. Degradation products (20–50 kDa) increase in strong positive samples. (**B**) *P. gingivalis* gingipain immunoblot showing bacterial protease in oral rinse samples. Lanes 1–8: patients with cancer with increasing test positivity; lane 9: healthy control.

The concurrent detection of gingipains, fragmented MMP-8, and elevated MMP-9 activation is consistent with the proposed proteolytic cascade, in which gingipains activate and fragment MMP-8, subsequently leading to MMP-9 activation and ultimately to tissue destruction (Figure 6).

## 4. Discussion

This study investigated the association between *Porphyromonas gingivalis* gingipains and the activation of MMP-8 and MMP-9 in patients with cancer, while also evaluating the clinical utility of a chair-side aMMP-8 test applied to oral rinse samples for the rapid detection of active periodontal inflammation. The 91% aMMP-8 positivity rate, approximately threefold higher than estimates reported for the general population, suggests a high burden of active periodontal inflammation in this vulnerable cohort. Importantly, we demonstrate molecular co-localization of bacterial gingipains with fragmented MMP-8 and activated MMP-9, consistent with a bacteria-associated amplification mechanism.

The significant correlation between aMMP-8 and MMP-9 activation (*p* = 0.003), despite a modest R^2^ (0.097), reflects the multifactorial nature of protease regulation in cancer. Our Western blot evidence, showing progressive MMP-8 fragmentation (from 70–80 kDa to 45–65 kDa bands), paralleling the gingipain signal intensity, provides molecular evidence consistent with bacterial protease-mediated activation. This extends beyond previous in vitro observations by demonstrating that the cascade operates in vivo within the oral environment of patients with cancer [23].

*P. gingivalis* gingipain activates latent MMPs through direct proteolytic cleavage while simultaneously degrading tissue inhibitors of metalloproteinases (TIMPs), creating a dual mechanism for amplifying tissue destruction. Our finding that gingipain presence correlates with both MMP-8 fragmentation patterns and MMP-9 activation suggests these bacterial proteases orchestrate a coordinated proteolytic response, although other host- and treatment-related factors may also contribute. This cascade mechanism may partly explain why patients with cancer experience accelerated periodontal deterioration despite treatment [5,6].

The chair-side aMMP-8 test demonstrated the ability to detect active periodontal inflammation within seven minutes, offering clear clinical utility. The test’s 91% positivity contrasts with the 69.5% periodontitis prevalence reported using conventional assessment in colorectal cancer [2], indicating that aMMP-8 detects active inflammatory processes that may precede or coexist with clinically diagnosed periodontitis. This aligns with the successful implementation of aMMP-8 chair-side test in non-dental settings, including ophthalmology [24], and pancreatic surgery clinics [25], and further suggests that the prevalence of periodontal disease among patients with cancer may be under-reported. Our finding that aMMP-8 correlates more strongly with oral hygiene indices (OHI-S and VPI) than periodontal parameters (PPD and GI) indicates that the test detects early inflammatory responses to bacterial biofilm. This early detection capability is particularly valuable in patients with cancer, where treatment-induced immunosuppression can accelerate progression from gingivitis to periodontitis. The observed dose–response relationship between visual chair-side test categories and quantitative aMMP-8 levels supports visual interpretation when digital readers are unavailable.

The lack of correlation between total MMP-8 and disease severity suggests that only the activated, collagenolytic form provides diagnostic value. This distinction is crucial: while total MMP-8 includes latent and TIMP-bound forms, aMMP-8 specifically reflects neutrophil degranulation and bacterial activation, the actual drivers of tissue destruction [26]. This biological specificity explains why aMMP-8, but not total MMP-8, differentiates between visual positivity categories and correlates with clinical parameters. Previous studies support this interpretation. Brandt et al. [27] found that total MMP-8 failed to reflect periodontal inflammation in patients with cancer, while aMMP-8 provided meaningful diagnostic information. However, compartment-specific differences exist; serum total MMP-8 and MMP-9 associate with systemic inflammation in colorectal cancer and renal cancer [28,29,30,31,32], highlighting the importance of sampling site.

The high periodontal disease burden observed in this cohort (mean PPD, 4.01 mm; 91% aMMP-8 positivity) likely reflects both cancer-related vulnerability and oral inflammatory processes. The gingipain-MMP cascade may contribute to cancer progression through multiple mechanisms, including enhanced tumor invasion via MMP-9 activation [33], disruption of epithelial barriers that facilitates bacterial translocation, and sustained inflammatory signaling through NF-κB pathways. *P. gingivalis* promotes oral squamous cell carcinoma invasion specifically through MMP-9 induction [23], while other proteases related to periodontal pathogens have been detected within tumor tissues [34,35,36,37]. The association between periodontitis and cancer risk may partially reflect this proteolytic mechanism [38,39]. Our findings suggest routine aMMP-8 screening could identify at-risk patients for targeted intervention, potentially reducing both local tissue destruction and systemic inflammatory burden during cancer treatment.

Integrating aMMP-8 chair-side test into routine oncology protocols could enable early detection and management of periodontal inflammation. Baseline screening at the time of cancer diagnosis would identify high-risk patients requiring immediate intervention, followed by monthly monitoring during chemotherapy or radiotherapy to detect treatment-induced oral changes. The visual test categories provide practical intervention thresholds: low positive results may prompt enhanced oral hygiene instruction, medium positive findings may indicate professional cleaning, and strong positive results may warrant comprehensive periodontal therapy, potentially including MMP inhibitors. The 20 ng/mL cutoff applied in this study represents an established optimal threshold [40,41,42,43]. Patients with head and neck cancer require particularly vigilant monitoring, given the direct effects of radiation on oral tissues as well as the indirect effects of chemotherapy [44,45,46,47,48,49,50,51,52,53,54,55]. The test’s non-invasive collection method, minimal patient burden, and immediate results address practical limitations frequently encountered in this population, where fatigue and treatment-related oral complications often prevent completion of traditional periodontal assessments. This streamlined approach ensures that even the most compromised patients receive appropriate periodontal screening and timely intervention, potentially reducing both local tissue destruction and systemic inflammatory burden during cancer treatment.

Several limitations should be acknowledged. This cross-sectional design precludes causal inference regarding the role of the gingipain-MMP cascade in cancer progression or treatment outcomes. All mechanistic interpretations should therefore be considered as associations requiring confirmation through longitudinal and interventional studies. The absence of an internal parallel control group is a significant limitation; the healthy control data for quantification of aMMP-8, tMMP-8, and MMP-9 activation were obtained from a separate study [15,21,22] conducted in a different population and clinical context, which limits the strength of direct comparisons. Among potential confounders, oral hygiene practices (brushing frequency) and smoking status were collected and reported descriptively (Table 1) but were not included as covariates in regression models due to limited variability (87.6% brushed twice daily; 93.3% were non-smokers). Cancer type and treatment modality were also recorded (Table 1). The absence of a parallel internal control group and incomplete data for covariates, including xerostomia, nutritional status, BMI, and recent antibiotic use, precluded adjustment for these factors, which may have contributed to variability in biomarker measurements. While the heterogeneous cancer types and treatment modalities introduce variability, this diversity enhances generalizability across oncology populations. The Western blot analysis was performed qualitatively without housekeeping protein loading controls, which limits quantitative interpretation. In vitro digestion experiments using recombinant MMP-8 with purified gingipains are needed to confirm cleavage specificity and validate the observed fragmentation patterns. The modest R^2^ values (0.097 for MMP-9 activation, 0.100 for OHI-S, and 0.067 for VPI) observed may reflect the biological complexity of protease regulation in cancer; however, they also highlight limited explanatory power, indicating that additional unmeasured host and microbial factors likely contribute to MMP activation beyond the pathways identified in this study.

Future studies should include subgroup analysis of different cancer types, gingipain-specific activity assays (e.g., using Rgp/Kgp-specific chromogenic substrates), and selective gingipain inhibition experiments to directly confirm the functional role of gingipains in the observed MMP fragmentation and activation, along with a confirmatory activity-based assay for MMP-8 activation.

In addition, studies can include well-matched, concurrently recruited healthy and periodontitis-only control groups processed under identical conditions, systematically adjust for relevant covariates in multivariate models, and employ longitudinal designs to track the dynamics of this cascade throughout cancer treatment trajectories, thereby enabling evaluation of whether periodontal intervention targeting this mechanism could influence cancer outcomes. Integrated multi-omics approaches, combining microbial profiling, protease activity mapping, and immune response characterization, would provide deeper mechanistic insights into the bacterial–host interactions driving tissue destruction. Ultimately, randomized trials testing routine aMMP-8 screening and cascade-targeted interventions within standard oncology care protocols are needed to establish clinical utility and determine whether disrupting this proteolytic cascade improves both oral health and cancer prognosis.

## 5. Conclusions

This study provides clinical evidence of an association between a gingipain-mediated proteolytic cascade and the activation of MMP-8 and MMP-9 in the oral cavity of patients with cancer. The 91% aMMP-8 positivity rate, combined with molecular evidence of gingipain co-localization with fragmented MMP-8 and activated MMP-9, suggests that bacterial proteases may contribute to host tissue destruction through the coordinated activation of MMPs. This mechanism may represent a targetable link between periodontal pathogens and the amplified inflammatory burden experienced by patients with cancer. The aMMP-8 chair-side test’s ability to detect this cascade within seven minutes offers immediate clinical utility. The clear dose–response relationship between visual categories and cascade intensity provides actionable thresholds for intervention: from enhanced hygiene instruction to comprehensive periodontal therapy. Integration into routine oncology protocols could allow early identification and disruption of this proteolytic cascade, potentially guiding interventions to reduce local tissue destruction and systemic inflammation; however, causal effects remain to be established. Beyond serving as a rapid screening biomarker, the gingipain-MMP cascade may represent a therapeutic target. Future interventions could include specific gingipain inhibitors, MMP modulators, or targeted antimicrobial approaches. This dual utility, both a diagnostic biomarker and a therapeutic target, positions the cascade as a key consideration in comprehensive cancer care.

## Figures and Tables

**Table 1:** Patients’ characteristics.

Characteristic	*n* (%)
**Demographics**	
Age, years (mean ± SD)	54.8 ± 12.3
Female sex	66 (74.2)
**Frequency of home tooth brushing**	
Twice in a day	78(87.6)
Once in a day	11 (12.4)
None in a day	0 (0)
**Smoking Status**	
Non-smoker	83 (93.3)
Current smoker	6 (6.7)
**Primary Cancer Site**	
Breast	36 (40.5)
Nasopharyngeal	34 (38.2)
Colorectal	11 (12.4)
Lymphoma	4 (4.5)
Cervical	2 (2.2)
Lung	1 (1.1)
Renal	1 (1.1)
**Cancer Location**	
Head and Neck	34 (38.2)
Non-Head and Neck	55 (61.8)
**Treatment Status**	
Chemotherapy	40 (44.9)
Combined chemoradiotherapy	18 (20.2)
Pre-treatment	11 (12.4)
Hormone therapy	8 (9.0)
Maintenance	8 (9.0)
Radiotherapy alone	3 (3.4)
Immunotherapy	1 (1.1)

**Table 2:** Periodontal Clinical Parameters and Biomarker Analysis.

Parameter	Value
**Clinical Periodontal Indices (mean ± SD)**	
Probing pocket depth (mm)	4.01 ± 1.32
Oral hygiene index-simplified (OHI-S)	3.08 ± 0.90
Visible plaque index (VPI)	2.53 ± 0.59
Gingival index (GI)	0.64 ± 0.46
**aMMP-8 Chair-side test Visual Results, *n* (%)**	
Negative	8 (9.0)
Positive (total)	81 (91.0)
- Low positive	46 (51.7)
- Medium positive	27 (30.3)
- Strong positive	8 (9.0)
**Biomarker Concentrations (mean ± SD)**	
aMMP-8 ORALyzer (ng/mL)	27.04 ± 10.55
Total MMP-8 ELISA (ng/mL)	46.38 ± 54.14
MMP-9 activation (%)	29.15 ± 28.42

## Data Availability

The data related to this study are available from the authors upon reasonable request.
